# Rheology-Informed Working Thresholds for HME/FDM Processability in a PVA–Sorbitol–Paracetamol Model System

**DOI:** 10.3390/pharmaceutics18070791

**Published:** 2026-06-27

**Authors:** Sofiya Ilieva, Dilyana Georgieva, Valentina Petkova, Milen Dimitrov

**Affiliations:** 1 Department of Pharmaceutical Technology and Biopharmacy, Faculty of Pharmacy, Medical University of Sofia, 2 Dunav Str., 1000 Sofia, Bulgaria; sofiailieva91@gmail.com (S.I.); dgeorgieva@pharmfac.mu-sofia.bg (D.G.); 2 Department of Organization and Economics of Pharmacy, Faculty of Pharmacy, Medical University of Sofia, 2 Dunav Str., 1000 Sofia, Bulgaria; vpetkova@pharmfac.mu-sofia.bg

**Keywords:** hot-melt extrusion, fused deposition modeling, pharmaceutical 3D printing, temperature-ramp rheology, complex viscosity, processability, polyvinyl alcohol, quality by design

## Abstract

**Background/Objectives**: Oscillatory rheology is widely used in hot-melt extrusion (HME) and fused deposition modeling (FDM), but its translation into compact formulation-screening criteria remains limited. This study re-analyzed an existing PVA–sorbitol–paracetamol dataset to derive rheology-informed working thresholds for HME/FDM processability. **Methods**: Temperature-ramp oscillatory rheology was used to extract formulation-level descriptors: process-temperature complex viscosity (|η*|) at 185 and 200 °C, Processing Window Fraction (PWF) within the 0.8–10 kPa·s corridor, and crossover-related temperature information. These descriptors were interpreted against empirical extrusion at 200 °C and printing at 185 °C. **Results**: S1.25 and S1.5 showed rheological behavior compatible with successful extrusion, whereas S1.75 showed pronounced softening consistent with over-plasticization and process failure. Paracetamol further reduced complex viscosity while maintaining processability in P5–P15. The lowest successful process-temperature viscosity values, observed for P15, supported working thresholds of approximately 0.460 kPa·s at 200 °C and 0.899 kPa·s at 185 °C. PWF complemented thresholding by describing the practical temperature flexibility of each formulation. **Conclusions**: Process-temperature |η*|, PWF, and crossover-informed interpretation provided a compact, formulation-specific screening framework for this PVA-based HME/FDM model system. The proposed thresholds are operational derivation outputs and require prospective external confirmation.

## 1. Introduction

Fused deposition modeling (FDM) has emerged as one of the most attractive additive manufacturing technologies for the preparation of personalized dosage forms because it combines geometric flexibility with the established pharmaceutical logic of hot-melt extrusion (HME). In this setting, the formulation must first be converted into a filament with sufficient mechanical integrity and melt processability and must then be reheated and deposited reproducibly through the printer nozzle. Rheology therefore occupies a central position in HME/FDM development, as it links formulation composition to melt flow, process temperature selection, and, ultimately, manufacturing feasibility [1,2,3,4].

Among the available rheological tools, oscillatory measurements are particularly informative for polymer-based pharmaceutical systems. They provide access not only to complex viscosity, but also to the viscoelastic balance between storage and loss moduli and to temperature-dependent transitions relevant to processing. Temperature-ramp experiments are especially useful in semi-crystalline systems because they help identify the region in which viscous behavior becomes dominant and the material enters a practically relevant processing domain [5,6]. In pharmaceutical formulation work, however, such outputs are often interpreted descriptively rather than translated into compact operational criteria that can support early go/no-go decisions.

In temperature-ramp oscillatory rheology, the relative evolution of G′ and G″ provides information on the transition from predominantly elastic to predominantly viscous behavior. The G′ = G″ crossover can be used as an operational crossover-related transition point, particularly in polymer systems where thermal softening or melting-related changes move the material toward viscous flow. Although this crossover should not be overinterpreted as a universal thermodynamic melting point, it provides a useful rheological anchor for assessing whether the selected processing temperatures are sufficiently positioned within a flow-dominated regime.

This translational gap matters in practice. During formulation screening, researchers and developers often need decision-relevant answers before investing further effort into larger-scale extrusion trials, printability testing, or downstream product characterization. The literature already supports the broader value of rheology for assessing HME processability, polymer–drug interactions, and FDM printability, including in pharmaceutical filament systems [5,6,7,8,9,10,11,12]. At the same time, most studies stop short of converting temperature-dependent rheological behavior into practically interpretable working thresholds that can be used as compact formulation-screening aids.

Our previous work established the experimental formulation landscape of a partially hydrolyzed PVA-based system plasticized with sorbitol and loaded with paracetamol, including successful and unsuccessful processing outcomes, thermal and solid-state characterization, and empirical printability evidence [13]. The present study does not re-report that formulation-based paper; instead, it extracts a secondary rheology-informed analytical framework from the same experimental platform. It therefore builds directly on the earlier formulation study while addressing a different, explicitly operational question. In particular, we focused on process-temperature complex viscosity, a Processing Window Fraction (PWF), and crossover-anchored temperature offsets as compact formulation-level descriptors.

This positioning is consistent with broader efforts to integrate pharmaceutical 3D printing with QbD-guided formulation design and established HME process science [14,15,16]. It also builds on rheology-based assessment of molten drug–polymer dispersions, including acetaminophen-containing systems, as well as FDM-oriented process modulation, printability enhancement, and wider pharmaceutical 3D-printing perspectives relevant to formulation development [17,18,19,20,21,22,23,24].

Importantly, the present work is not intended to claim universal predictive validity across polymers, plasticizers, APIs, and equipment configurations. Instead, it is designed to derive working, formulation-specific thresholds and heuristics from a defined model system and to examine whether these descriptors align with the observed extrusion and printing outcomes. This distinction is deliberate: in early-stage pharmaceutical process development, a transparent and well-bounded operational framework is often more valuable than an overstated predictive claim.

Accordingly, the aim of this study was to derive rheology-informed working thresholds for HME/FDM processability in a PVA–sorbitol–paracetamol model system by relating temperature-dependent viscoelastic behavior to empirical extrusion at 200 °C and printability at 185 °C. A secondary objective was to integrate these descriptors into a compact processability map that could support formulation screening and process interpretation in a quality-by-design (QbD)-oriented context. The practical value of the present work is that it moves temperature-ramp rheology one step beyond description: into a compact screening framework anchored to empirical HME/FDM outcomes.

## 2. Materials and Methods

### 2.1. Study Design and Analytical Scope

This work represents an exploratory derivation study based on an existing experimental dataset obtained from a PVA-based filament system developed for HME and subsequent FDM. The objective of the present analysis was not to reproduce the full experimental formulation study, but to extract compact rheology-informed descriptors that could be interpreted against empirically observed extrusion and printability outcomes within the same model system.

### 2.2. Materials and Formulation Set

Paracetamol was used as a model active pharmaceutical ingredient (API) and was purchased from Hebei Jiheng Pharmaceutical Co., Ltd. (Hengshui, China). Polyvinyl alcohol (PVA; molecular weight 31,000–50,000 g/mol; 87–89% degree of hydrolysis) was obtained from Acros Organics (Geel, Belgium) and used as the polymer matrix. Sorbitol was acquired from DHW (Dessau-Roßlau, Germany) and used as hydrophilic plasticizer. A commercial PVA filament from Shenzhen Lankeda Technology Co., Ltd. (Shenzhen, China) served as practical reference material. All materials were used as received.

The formulation codes reflect the composition of the placebo and drug-loaded systems. S1.25, S1.5, and S1.75 denote placebo PVA–sorbitol formulations with PVA:sorbitol mass ratios of 10:1.25, 10:1.5, and 10:1.75, respectively. After S1.5 had been identified as the most suitable placebo base for further processing, drug-loaded formulations were prepared using the S1.5 excipient ratio and nominal paracetamol loadings of 5%, 10%, and 15% *w*/*w*, denoted as P5, P10, and P15, respectively [13].

### 2.3. Experimental Provenance and HME/FDM Processing Conditions

The present analysis re-used temperature-ramp oscillatory rheology data generated from placebo and drug-loaded formulations that had already been processed experimentally by HME and, where applicable, by FDM printing. In the underlying formulation study, placebo filaments were produced by HME at 200 °C, and printability was assessed at 185 °C using simple printed discs for placebo screening and tablets for drug-loaded formulations. The same experimental workflow provided empirical observations of successful extrusion for S1.25, S1.5, P5, P10, and P15; successful printing evidence for S1.5 and the drug-loaded formulations at 185 °C; and unsuccessful processing for S1.75 [13].

Extrusion was performed using a 16 mm single-screw filament extruder (Wellzoom, Shenzhen, China) equipped with two heating zones and a 1.80 mm die. To improve feed uniformity and partly compensate for the limited mixing capacity of the single-screw configuration, all samples were granulated before feeding into the hopper. Extrusion was performed at 200 °C in both heating zones and at a screw speed of 10 rpm. The system included a dual-roller puller for filament diameter adjustment and a single-reel winder for filament collection. The target filament diameter was 1.75 mm and was monitored using a digital caliper.

Mass flow rate was not recorded in the original experimental workflow. This is considered a process-characterization limitation of the present secondary analysis, because the underlying study was conducted in a proof-of-concept filament-production mode rather than under gravimetrically monitored continuous manufacturing conditions.

### 2.4. Rheological Measurements

Rheological measurements were performed using a stress-controlled rotational rheometer AR-G2 (TA Instruments, New Castle, DE, USA) equipped with electrically heated parallel plates. A 25 mm plate diameter and a fixed gap of 1 mm were used. Before testing, the samples were heated to 170 °C and compressed into 22 mm slugs using a Carver hydraulic press (Carver Inc., Wabash, IN, USA).

Preliminary strain-sweep tests were performed for all samples at 185 °C and 1 Hz over a strain range of 0.01–1000% to identify the linear viscoelastic region (LVR). The subsequent temperature-ramp measurements were performed at a constant strain amplitude of 0.5% and a constant frequency of 1 Hz. Samples were first equilibrated at 210 °C for 1 min and then cooled from 210 to 150 °C at 5 °C/min. Storage modulus (G′), loss modulus (G″), and complex viscosity (|η*|) were recorded as functions of temperature.

The preliminary strain-sweep experiments were used to identify the LVR and to select the strain amplitude for the temperature-ramp measurements. They were not treated as a replicated yield-stress or yield-point dataset for quantitative threshold derivation. The temperature-ramp measurements were used to characterize the temperature-dependent transition from predominantly elastic to predominantly viscous behavior and to estimate formulation-level processing descriptors.

### 2.5. Derived Rheological Descriptors

For the purposes of the present analytical study, the following per-formulation descriptors were extracted or derived: (i) complex viscosity at process temperature, expressed as |η*| at 185 °C and 200 °C, corresponding to the empirical printing and extrusion set-points used in the underlying experiments; (ii) Processing Window Fraction (PWF), defined as the fraction of the 150–210 °C interval within which |η*| remained within the practical viscosity band of 0.8–10 kPa·s; (iii) operational crossover-related temperature anchor, defined as the temperature at which G′ = G″ when such crossover was observable within the experimental temperature range; (iv) temperature offsets relative to this crossover-related anchor, expressed as ΔT values between the empirical process temperature and the rheological crossover anchor, where applicable; and (v) normalized viscosity descriptors, used to interpret the effect of API loading relative to the placebo matrix.

### 2.6. Outcome Definitions

Two empirical binary outcomes were used in the analytical mapping: Extrude@200, defined as successful filament extrusion at 200 °C; and Print@185, defined as successful printing at 185 °C for formulations for which direct printing evidence was available in the underlying dataset. These outcomes were not generated de novo for the present analysis but were inherited from the experimentally observed processing behavior of the analyzed formulations in the original formulation-development workflow [13]. Because direct placebo printing evidence was explicitly documented for S1.5, while S1.25 was mainly supported at the extrusion stage, printability-related interpretation was kept more cautious than extrusion-related interpretation.

### 2.7. Working Threshold Derivation and Statistical Handling

Single-predictor threshold derivation was performed using process-temperature complex viscosity (|η*|) as the main candidate descriptor for each empirical outcome. The thresholding procedure was intentionally simple and exploratory. Operational cut-off values were identified from the derivation dataset and interpreted as working thresholds for this specific model system.

Procedurally, the thresholding logic followed three steps: first, extraction of per-formulation |η*| values at the empirical process temperatures; second, ordering of formulations against the binary processing outcomes; and third, retention of the lowest successful observed values as operational boundaries. The auxiliary cut-off sheet was used as a consistency check rather than as the primary source of evidentiary weight.

The rheological dataset available for this secondary analysis consisted of one temperature-ramp measurement per formulation. Therefore, the derived process-temperature viscosity values, PWF values, and crossover-related descriptors were treated as descriptive formulation-level outputs. Means, standard deviations, confidence intervals, and inferential statistical comparisons were not calculated, because such calculations would imply replication that was not present in the dataset.

Because the analytical dataset was limited in size and compositional breadth, the proposed thresholds were treated as exploratory derivation outputs rather than externally validated predictive criteria. Their role in the present manuscript is therefore operational rather than confirmatory.

### 2.8. Processability Mapping and Complementary Interpretation

To avoid over-reliance on a single rheological variable, process-temperature |η*| was interpreted together with PWF and crossover-related temperature information. This combined reading was used to build a compact rheology-informed processability map intended to support practical early-stage HME/FDM formulation screening. The resulting framework was designed as a decision aid for this model system, not as a substitute for full downstream formulation evaluation.

### 2.9. Microscopy and Printability Evidence

In the underlying experimental dataset, printability was additionally supported by the successful preparation of 3D-printed objects and tablets, and by light microscopy and scanning electron microscopy of representative printed units [13]. These observations were not re-analyzed quantitatively in the present work, but were used as contextual empirical support for the printability outcome definition.

### 2.10. Analytical Restraint

The present framework was intentionally designed as a transparent decision-support tool for early-stage formulation screening, not as a replacement for full product-performance testing or prospective process validation. All threshold-related outputs were therefore interpreted conservatively and within the bounds of the analyzed PVA-based HME/FDM system.

## 3. Results and Discussion

**Figure 1:**
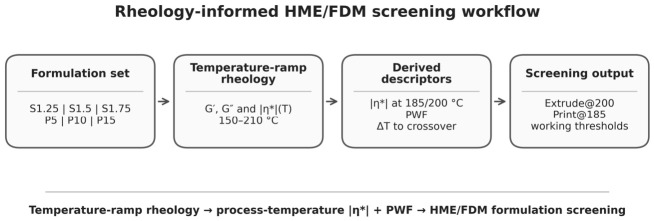
Conceptual workflow of the analytical study. Temperature-ramp oscillatory rheology was used to derive formulation-level descriptors, including crossover-related temperature information, process-temperature complex viscosity, and Processing Window Fraction (PWF). These descriptors were then interpreted against empirical extrusion at 200 °C and printing at 185 °C.

The analytical workflow used to derive and interpret the rheology-informed descriptors is summarized in Figure 1.

### 3.1. Temperature-Dependent Viscoelastic Behavior of the Placebo and Drug-Loaded Formulations

Temperature-ramp oscillatory rheology clearly differentiated the placebo formulations and provided a physically meaningful basis for interpreting their processability. Among the placebo systems, S1.25 and S1.5 displayed viscoelastic profiles broadly comparable to the commercial PVA filament. In these formulations, observable G′ = G″ crossover points were retained within the measured temperature interval, indicating an operational crossover-related transition toward more viscous, flow-dominated behavior. In contrast, S1.75 showed markedly lower modulus values and a substantially softened rheological profile, consistent with over-plasticization. Its crossover-related transition was not observed within the measured interval, supporting the interpretation that this formulation had moved outside the practically useful viscoelastic balance for stable filament formation. These observations are aligned with the empirical processing behavior reported previously, where S1.25 and S1.5 were successfully extruded, S1.5 showed direct placebo printability evidence, and S1.75 yielded poor filament quality and was not suitable for practical printing [13].

The addition of paracetamol shifted the rheological behavior further toward a more fluid regime. In the drug-loaded subset, increasing API load was accompanied by lower modulus values and lower process-temperature complex viscosity, supporting the interpretation that paracetamol contributed an additional plasticizing effect within the investigated concentration range. The crossover-related transition of the drug-loaded formulations fell below the lower bound of the measured temperature interval, which is consistent with a further shift in the PVA–sorbitol matrix toward viscous behavior. Importantly, this shift remained compatible with successful processing at the selected set-points and therefore did not indicate loss of processability within the studied formulation window. The crossover information therefore complements the process-temperature viscosity reading by providing a physical anchor for the elastic-to-viscous transition.

**Figure 2:**
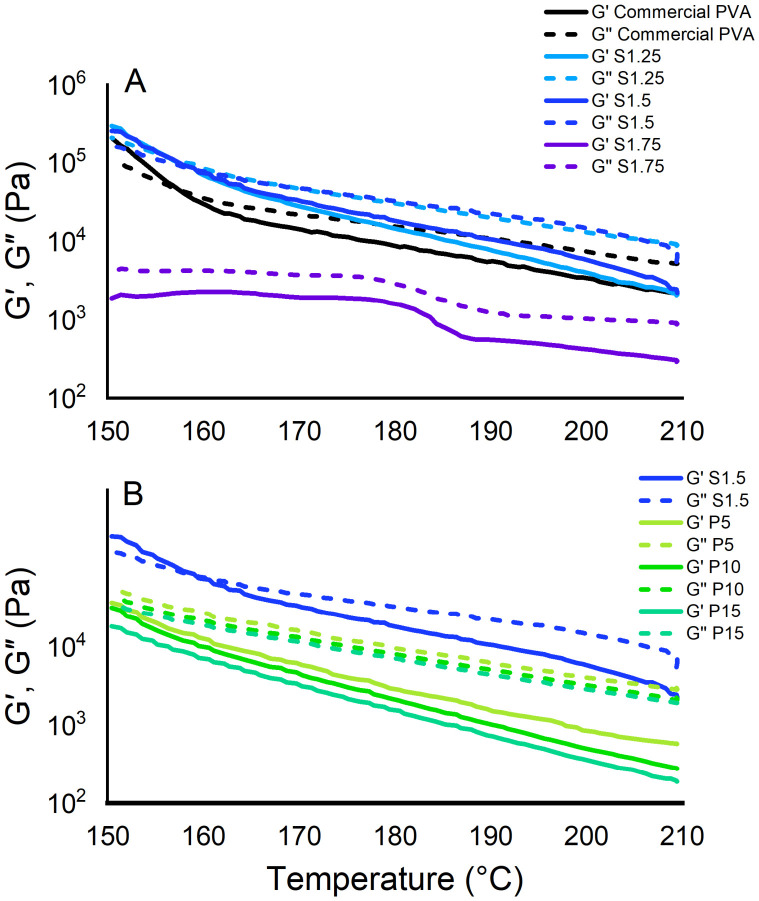
Temperature-dependent storage modulus (G′) and loss modulus (G″) profiles. (**A**) Placebo formulations compared with the commercial PVA filament. (**B**) Optimized placebo formulation S1.5 compared with the drug-loaded formulations P5, P10, and P15. Colors denote formulations, while solid and dashed lines denote G′ and G″, respectively.

The corresponding temperature-dependent G′ and G″ profiles are shown in Figure 2.

### 3.2. Process-Temperature Complex Viscosity and Empirical Processability

When the dataset was re-read specifically at the empirical process temperatures, |η*| emerged as the most practically intuitive descriptor. The placebo formulations S1.25 and S1.5 occupied a higher-viscosity region compatible with successful extrusion, whereas S1.75 fell into a markedly lower-viscosity regime associated with process failure. Direct printing evidence at 185 °C was available for S1.5 and the drug-loaded formulations, which occupied viscosity ranges that remained operationally compatible with the selected printing conditions. This pattern supports the use of process-temperature |η*| as a compact screening cue while preserving the distinction between direct printing evidence and more cautious printability inference.

This reading is useful because it translates temperature-ramp rheology into a parameter that can be applied directly during formulation development. Rather than interpreting the entire rheological curve qualitatively, one may ask whether a formulation occupies a practically acceptable viscosity region at the intended process set-point. In the present model system, that process-temperature perspective proves more decision-relevant than any single rheological transition considered in isolation.

**Figure 3:**
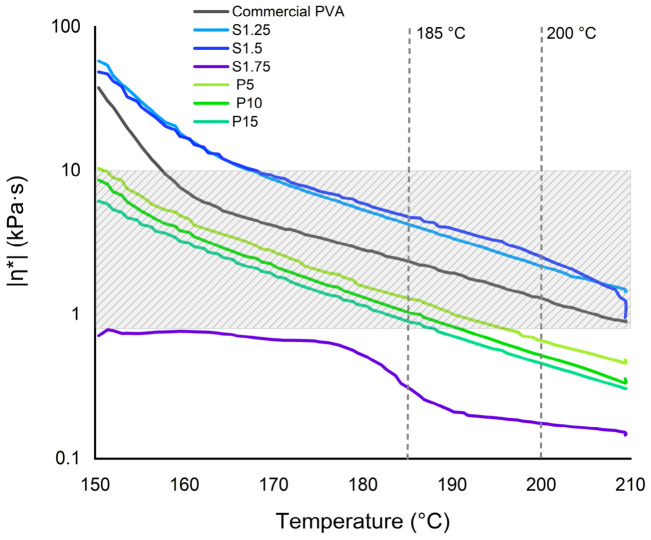
Complex viscosity (|η*|) as a function of temperature for placebo and drug-loaded formulations. The shaded/hatched band indicates the practical HME/FDM viscosity corridor of 0.8–10 kPa·s used for PWF calculation, and the vertical dashed lines mark the empirical process temperatures of 185 °C and 200 °C.

The process-temperature viscosity reading is further illustrated in Figure 3.

### 3.3. API-Related Viscosity Reduction and Mixture Behavior

Normalized viscosity trends further supported the interpretation that paracetamol remained compatibly incorporated within the matrix across the investigated concentration range. As API loading increased from P5 to P15, normalized viscosity decreased rather than rebounded, which is consistent with continued rheological softening rather than an obvious saturation-related reversal. Within the bounds of the present model system, this behavior supports the view that the solubilization capacity of the matrix had not yet been exceeded at the highest investigated drug load.

This point is mechanistically useful because it links rheological behavior not only to processing feasibility, but also to mixture behavior within the polymer matrix. At the same time, the interpretation should remain formulation-specific. Rheology can provide a strong indirect indication, but it does not replace orthogonal confirmation where solid-state or phase-behavior claims are central.

### 3.4. Derivation of Working Thresholds and Process-Window Descriptors

To move from descriptive rheology toward operational decision support, process-temperature complex viscosity (|η*|) was used to derive formulation-specific working thresholds against the empirical outcomes Extrude@200 and Print@185. Within the derivation dataset, process-temperature viscosity separated successful and unsuccessful formulations in a manner that supported practical threshold extraction for this model system. This separation is useful, but it should be interpreted as internal to the derivation dataset rather than as evidence of external predictive validation.

The 0.8–10 kPa·s interval was used as a practical viscosity corridor for calculating the Processing Window Fraction. This corridor should not be interpreted as a universal acceptance range for all HME/FDM systems. Rather, it was used as an operational window for the present analysis, informed by the practical viscosity order of magnitude discussed in HME and the polymer melt-processability literature and refined according to the observed behavior of the present formulation set [5,17,25,26,27]. Previous reports have discussed practical viscosity ranges around 1–10 kPa·s for polymers relevant to HME, while other work emphasizes the practical relevance of high-viscosity limitations and an upper boundary around 10 kPa·s for processability [17,25,26,27]. In the present model system, the upper boundary of 10 kPa·s was retained as a practical high-viscosity limit for stable extrusion/printing interpretation, whereas the lower boundary of 0.8 kPa·s was selected as a data-informed boundary close to the lowest successful printing-relevant viscosity observed in the dataset. Thus, 0.8 kPa·s should be interpreted as a formulation-specific operational lower boundary, not as a universal literature-derived limit.

The distinction between the PWF corridor and the process-temperature thresholds is important. The PWF corridor describes how much of the measured 150–210 °C interval falls within a practically workable viscosity region. In contrast, the process-temperature thresholds were derived at the specific empirical set-points used in the underlying experiments. Working cut-offs of approximately 0.460 kPa·s at 200 °C and 0.899 kPa·s at 185 °C were obtained from the derivation set, corresponding to the lowest successful process-temperature viscosity values observed for P15. Thus, the PWF corridor and the individual process-temperature thresholds serve complementary but distinct purposes: PWF describes temperature-window breadth, whereas the thresholds describe boundary behavior at the selected extrusion and printing temperatures.

The threshold logic gained additional interpretive strength when viewed alongside the Processing Window Fraction. Unlike a single-temperature descriptor, PWF captures how much of the measured temperature interval remains within the practical viscosity corridor. In this sense, PWF functioned as a contextual descriptor of processing flexibility. Formulations with favorable empirical outcomes tended to remain within the practical viscosity corridor for a larger fraction of the tested temperature range, while the failed S1.75 formulation showed a collapsed or absent working window.

This combined interpretation is more informative than thresholding alone. A cut-off indicates where a formulation sits at a selected set-point, whereas PWF helps indicate how tolerant—or how fragile—that position may be with respect to temperature variation. The over-plasticized S1.75 formulation was analytically useful because it provided a negative boundary case against which the lower-viscosity region of the working space could be interpreted.

For clarity, the formulation set, empirical outcomes, and derived rheological descriptors are summarized in Table 1.

**Figure 4:**
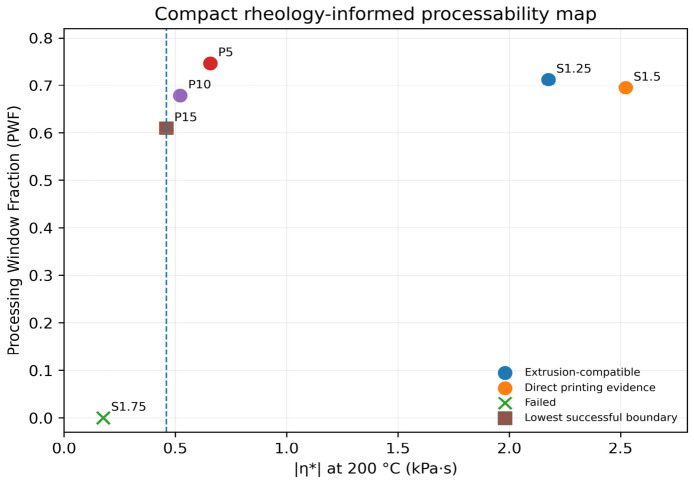
Compact rheology-informed processability map. Process-temperature |η*| at 200 °C is integrated with PWF to visualize how successful and unsuccessful formulations are positioned within the derivation dataset. Blue and orange circles denote placebo formulations associated with successful extrusion or direct printing evidence, respectively. Red and purple circles denote drug-loaded formulations with successful processing. The brown square denotes the lowest-viscosity successful boundary formulation, P15, while the green cross denotes the failed over-plasticized formulation, S1.75. The vertical blue dashed line indicates the process-temperature working threshold at 200 °C, derived from the lowest successful |η*| value observed for P15.

The combined descriptor space is visualized in Figure 4.

### 3.5. Crossover-Anchored Temperature Interpretation and Practical Use

The crossover-informed reading added a further layer of physical interpretability. In the placebo formulations where an operational G′ = G″ crossover-related transition remained observable within the measured interval, the empirical process temperatures were positioned above this rheological anchor. This supports the practical interpretation that successful processing in this PVA-based system required not merely entry into a viscosity-dominant regime, but sufficient temperature offset from the crossover-related transition to ensure stable material flow under the specific conditions of the single-screw extruder and FDM printer.

This point is particularly useful in a QbD-oriented context. It suggests that processability may be interpreted not only through absolute viscosity alone, but through a compact combination of (i) where the formulation sits relative to a practically acceptable viscosity band and (ii) how far the selected process temperature lies from the operational viscoelastic transition region. Framed in this way, the rheological readout becomes a structured formulation-development aid rather than a purely descriptive characterization output.

### 3.6. Practical Implications for HME/FDM Formulation Screening

From a formulation-development perspective, the present framework offers a compact way to reduce early-stage trial-and-error in HME/FDM development. Rather than relying exclusively on empirical extrusion and printing trials, developers may first ask three practical questions: does the formulation occupy a workable process-temperature viscosity region, does it retain a meaningful Processing Window Fraction, and is the selected process set-point plausibly positioned relative to the rheological crossover behavior? This does not eliminate the need for full downstream evaluation, but it can make early candidate triage more rational and transparent.

The approach may be particularly useful during pre-formulation and early process-development work, where only limited material is available and multiple polymer–plasticizer–API combinations need to be screened. In this context, temperature-ramp rheology can help identify formulations that are likely to be over-plasticized, too viscous for stable deposition, or insufficiently robust with respect to process-temperature variation. The framework therefore provides a practical bridge between rheological characterization and empirical HME/FDM decision-making.

The same logic may also support future continuous-manufacturing development. In a more advanced setting, rheology-derived descriptors such as process-temperature |η*| and PWF could be interpreted together with extruder torque, die pressure, filament diameter stability, residence time, mass flow rate, and printing performance. Such integration would be consistent with QbD-oriented process understanding and may help transform rheology from an offline characterization method into a formulation-screening and process-development tool.

The practical value of this approach is greatest when it is used with restraint. The framework is best viewed as a screening aid for this model system, not as a universal rheological law of HME/FDM processability. Its present strength lies in compactness, interpretability, and alignment with empirically observed outcomes within a defined pharmaceutical formulation space. For a formulation scientist, the framework is useful before the expensive part begins: committing material, instrument time, and downstream testing to candidates that may already sit outside a workable rheological window.

### 3.7. Limitations and Outlook

The limitations of the present study are not incidental; they define the proper use of the proposed framework. First, the manuscript should be read as a secondary analytical framework extracted from an already established experimental platform rather than as a replacement for the original formulation study on which the dataset was built. Second, the available rheological dataset consisted of one temperature-ramp measurement per formulation. Therefore, the derived viscosity, PWF, and crossover-related descriptors were interpreted descriptively, without mean values, standard deviations, confidence intervals, or inferential statistical comparisons.

Third, no independent external validation set was available. The proposed thresholds should therefore be interpreted as exploratory working thresholds derived from a defined PVA–sorbitol–paracetamol model system, not as externally validated predictive criteria. The derivation dataset was also small and compositionally narrow, including one partially hydrolyzed PVA grade, one plasticizer, one model API, and one equipment configuration. Threshold portability across other polymers, plasticizers, APIs, drug loads, extruders, printers, or nozzle geometries remains to be established.

Fourth, the underlying experimental workflow was not designed as a gravimetrically monitored continuous-manufacturing study. Mass flow rate was not recorded, and the rheological descriptors could not be directly linked to torque, die pressure, residence time, filament diameter stability, or in-line process signals. These parameters should be incorporated in future studies to evaluate whether the proposed descriptors can support more advanced process monitoring and scale-up.

Fifth, the preliminary strain-sweep experiments were used to identify the linear viscoelastic region and to select the strain amplitude for temperature-ramp testing. They were not designed as a replicated yield-stress or yield-point dataset. Future work should therefore examine whether yield-related parameters, extensional behavior, melt strength, and filament mechanical performance provide additional predictive value beyond process-temperature |η*|, PWF, and crossover-informed interpretation.

Finally, the applicability of any HME/FDM framework remains constrained by the thermal stability of the API and excipients. The present model system was interpreted within the process temperatures and residence conditions of the underlying dataset. For thermolabile drugs or excipients, rheological suitability alone would not be sufficient; degradation, assay, impurity formation, solid-state stability, and product-performance testing would need to be evaluated before a formulation could be considered practically suitable for HME/FDM processing.

These limitations do not diminish the practical relevance of the present work; rather, they define its proper scope. The next logical step is prospective testing of the proposed descriptors across expanded formulation grids and additional polymer–API systems. Future studies may test whether PWF remains informative across other thermoplastic polymers, plasticizer systems, APIs, extrusion geometries, and printing conditions. Such work would clarify whether the present thresholds remain system-specific or whether parts of the framework may generalize more broadly as a rheology-informed HME/FDM screening strategy.

## 4. Conclusions

Temperature-dependent oscillatory rheology can be translated into practical, formulation-level decision aids for HME/FDM development when interpreted in a disciplined and application-oriented manner. In the present PVA–sorbitol–paracetamol model system, process-temperature complex viscosity, Processing Window Fraction, and crossover-informed temperature interpretation jointly provided a compact framework for reading extrusion and printability behavior against the rheological profile of each formulation.

Within the derivation dataset, these descriptors supported the extraction of working thresholds for the selected process temperatures and helped distinguish formulations associated with successful processing from the over-plasticized failed case. Importantly, these thresholds should be regarded as formulation-specific operational guides rather than externally validated universal criteria.

Taken together, the present study supports a more structured use of oscillatory rheology in early HME/FDM formulation screening. The framework is best used for formulation triage, process set-point interpretation, and disciplined pre-screening before further experimental iteration. Prospective validation across broader formulation spaces, additional polymer–API systems, and more fully monitored processing conditions will be required before wider generalization can be claimed.

## Figures and Tables

**Table 1:** Formulation set, empirical processing outcomes, and derived rheological descriptors. The table summarizes the empirical outcome coding used in the manuscript together with the formulation-level rheological descriptors that underpin the proposed working thresholds. Extrude@200 reflects successful filament extrusion at 200 °C, whereas Print@185 reflects direct printing evidence at 185 °C where such evidence is available in the underlying dataset. For S1.25, the label “cautious/indirect support” reflects extrusion-compatible rheology and successful extrusion, but not the same level of direct placebo printing evidence available for S1.5 and the drug-loaded formulations.

Formulation	Role	Extrude@200 °C	Print@185 °C	Crossover Observable	|η*| 200 °C (kPa·s)	|η*| 185 °C (kPa·s)	PWF	Interpretation
S1.25	Placebo	Yes	Cautious/indirect support	Yes	2.176	4.274	0.712	Extrusion-compatible higher-viscosity placebo
S1.5	Optimized placebo	Yes	Yes	Yes	2.523	4.822	0.695	Optimized placebo benchmark
S1.75	Over-plasticized placebo	No/poor filament quality	No	No/below range	0.176	0.314	0.000	Failed over-plasticized formulation; collapsed window
P5	Drug-loaded	Yes	Yes	No/below range	0.657	1.301	0.746	Workable API-containing formulation
P10	Drug-loaded	Yes	Yes	No/below range	0.522	1.040	0.678	Lower viscosity, still processable
P15	Drug-loaded	Yes	Yes	No/below range	0.460	0.899	0.610	Lowest-viscosity successful boundary

## Data Availability

The data supporting the findings of this study are included in the article and Supplementary Materials. Additional analysis files underlying the rheology-derived descriptors and threshold calculations are available from the corresponding author upon request.

## Supplementary Materials

The following supporting information can be downloaded at https://www.mdpi.com/article/10.3390/pharmaceutics18070791/s1: Table S1: per-formulation rheological descriptors and empirical outcomes; Table S2: interpolated process-temperature rheological descriptors at 185 and 200 °C; Table S3: Processing Window Fraction calculations and process-window status; Figure S1: auxiliary cut-off output; Figure S2: supplementary normalized-viscosity output; Figure S3: extended process-window profiles used for PWF calculation.
