# Supplementary material for: Rheology-Informed Working Thresholds for HME/FDM Processability in a PVA–Sorbitol–Paracetamol Model System
Source: Pharmaceutics. 2026 Jun 27;18(7):791. doi: 10.3390/pharmaceutics18070791 (PMC13416039; doi:10.3390/pharmaceutics18070791)
Supplement: Supplementary file 1 [file pharmaceutics-18-00791-s001.zip › pharmaceutics-4325651-supplementary.pdf]

Supplementary Materials

Rheology-Informed Working Thresholds for HME/FDM Processability in a PVA–Sorbitol–Paracetamol Model System

This supplementary file contains the source-derived and processed rheological descriptors, supplementary cut-off outputs, normalized-viscosity outputs, and extended process-window profiles used to support the analysis.

Table S1. Per-formulation rheological descriptors and empirical outcomes.

Formulation	Role	Crossover anchor (°C)	ΔT185 vs anchor (°C)	ΔT200 vs anchor (°C)	PWF	Extrude@200	Print@185
commercial PVA	Reference	157.5	27.5	42.5	0.881	Yes	Reference
S1.25	Placebo	156.5	28.5	43.5	0.712	Yes	Cautious / indirect support
S1.5	Optimized placebo	159.1	25.9	40.9	0.695	Yes	Yes
S1.75	Over-plasticized placebo	n.d.	n.d.	n.d.	0.000	No	No
P5	Drug-loaded	n.d.	n.d.	n.d.	0.746	Yes	Yes
P10	Drug-loaded	n.d.	n.d.	n.d.	0.678	Yes	Yes
P15	Drug-loaded	n.d.	n.d.	n.d.	0.610	Yes	Yes

Note. The operational crossover-related temperature anchor was defined as G′ = G″ when such crossover was observable in the experimental range. Print@185 for S1.25 is retained as cautious/indirect support, in line with the main manuscript.

Table S2. Interpolated process-temperature rheological descriptors at 185 and 200 °C.

Formulation	|η*|185 (kPa·s)	G′185 (kPa)	G″185 (kPa)	tanδ185	|η*|200 (kPa·s)	G′200 (kPa)	G″200 (kPa)	tanδ200
commercial PVA	2.361	6978.600	13093.000	1.876	1.302	3421.000	7430.667	2.172
S1.25	4.274	10.581	24.681	2.333	2.176	4.012	13.070	3.258
S1.5	4.822	13.878	26.934	1.941	2.523	5.830	14.740	2.528
S1.75	0.314	815.310	1795.400	2.202	0.176	416.467	1022.667	2.456
P5	1.301	2215.800	7867.400	3.551	0.657	849.067	4041.000	4.759
P10	1.040	1465.800	6367.400	4.344	0.522	499.067	3241.000	6.494
P15	**0.899**	1049.000	5547.700	5.289	**0.460**	356.767	2865.000	8.030

Note. Process-temperature descriptors were extracted at the empirical printing and extrusion set-points. Values for P15 corresponding to the lowest successful process-temperature viscosity are emphasized in bold.

Table S3. Processing Window Fraction calculations and process-window status.

Formulation	PWF	|η*|185 status	|η*|200 status	Processing interpretation

commercial PVA	0.881	Within 0.8–10	Within 0.8–10	Reference material; broad practical window
S1.25	0.712	Within 0.8–10	Within 0.8–10	Higher-viscosity placebo; extrusion-compatible
S1.5	0.695	Within 0.8–10	Within 0.8–10	Optimized placebo benchmark
S1.75	0.000	Below 0.8	Below 0.8	Collapsed/absent practical window
P5	0.746	Within 0.8–10	Below 0.8	API-loaded formulation; processable
P10	0.678	Within 0.8–10	Below 0.8	API-loaded formulation; lower viscosity, still processable
P15	0.610	Within 0.8–10	Below 0.8	Lowest-viscosity successful boundary

Note. PWF denotes the fraction of the 150–210 °C interval within which $|\eta^*|$ remained in the practical viscosity corridor of 0.8–10 kPa·s.

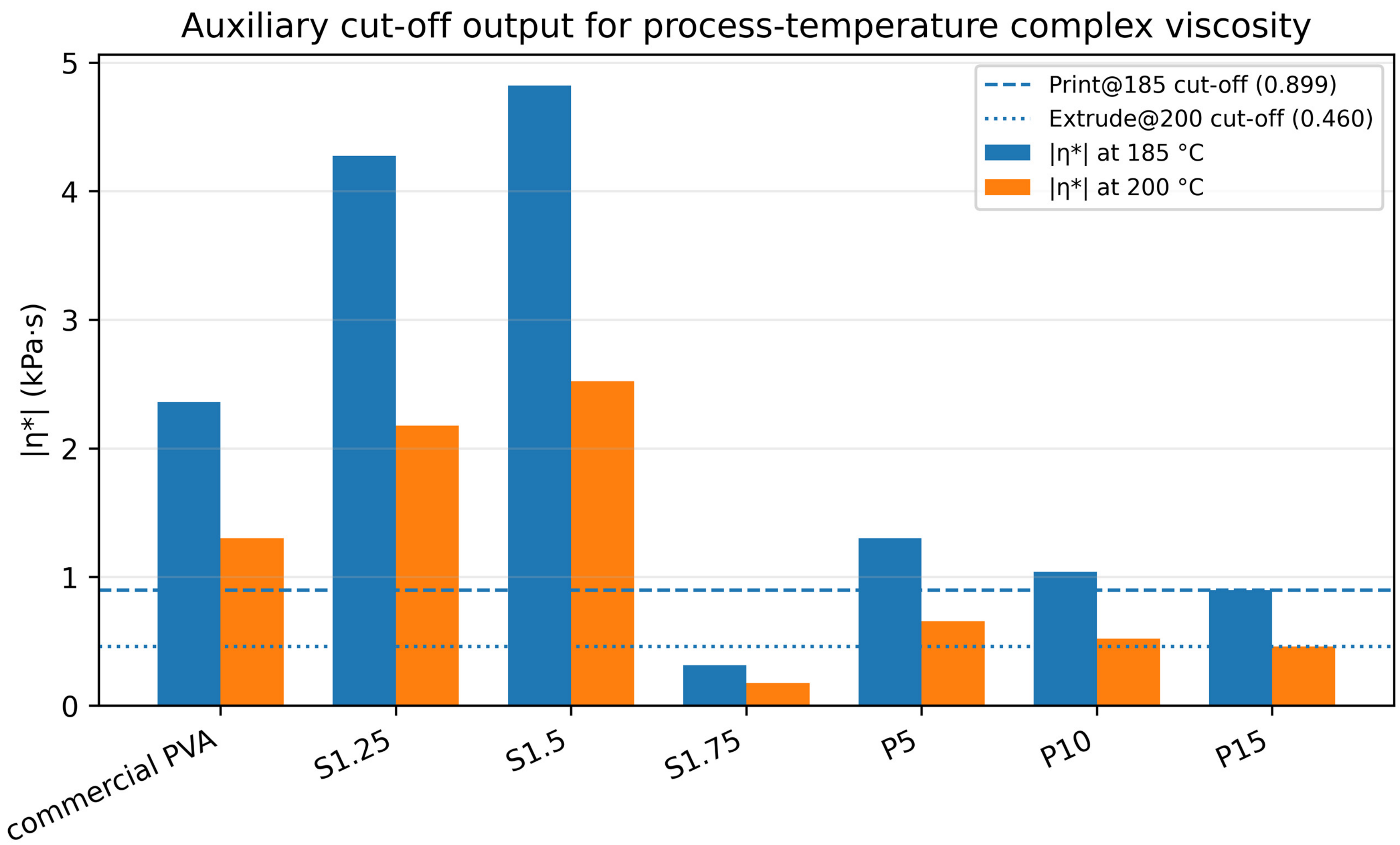

Figure S1. Auxiliary cut-off output. Process-temperature complex viscosity values are shown against the working cut-offs derived from the lowest successful values in the internal derivation dataset.

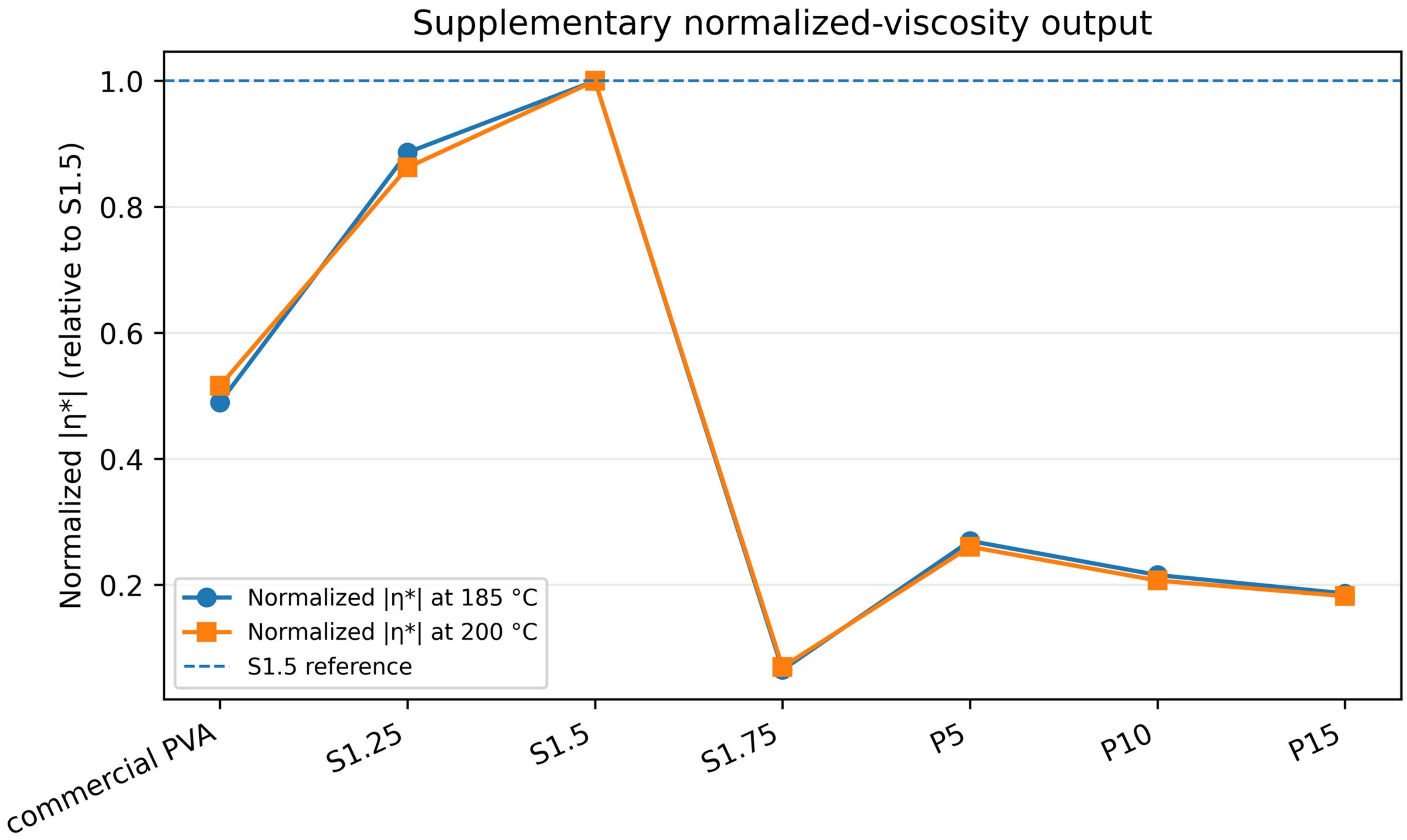

Figure S2. Supplementary normalized-viscosity output. Normalized complex viscosity values are reported relative to the S1.5 placebo benchmark at the corresponding process temperature.

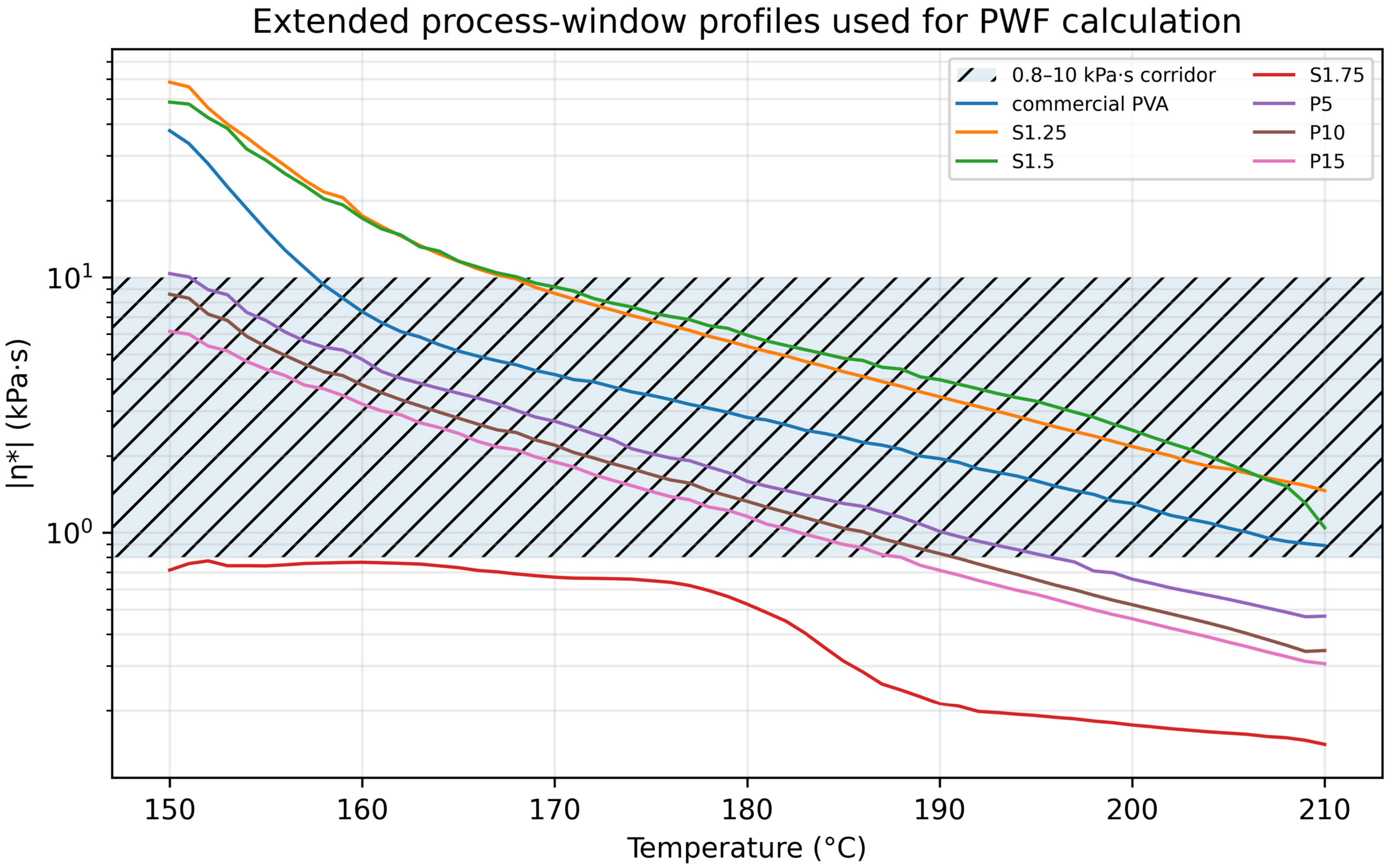

Figure S3. Extended process-window profiles used for PWF calculation. Integer-grid complex-viscosity profiles across 150–210 °C illustrate the portion of the temperature interval occupying the 0.8–10 kPa·s corridor.
